# Strongly Localized Image States of Spherical Graphitic Particles

**DOI:** 10.1155/2014/726303

**Published:** 2014-01-22

**Authors:** Godfrey Gumbs, Antonios Balassis, Andrii Iurov, Paula Fekete

**Affiliations:** ^1^Department of Physics and Astronomy, Hunter College at the City University of New York, 695 Park Avenue, New York, NY 10065, USA; ^2^Physics Department, Fordham University, 441 East Fordham Road, Bronx, NY 10458-5198, USA; ^3^Department of Physics and Nuclear Engineering, US Military Academy, 698 Mills Road, West Point, NY 10996, USA

## Abstract

We investigate the localization of charged particles by the image potential of spherical shells, such as fullerene buckyballs. These spherical image states exist within surface potentials formed by the competition between the attractive image potential and the repulsive centripetal force arising from the angular motion. The image potential has a power law rather than a logarithmic behavior. This leads to fundamental differences in the nature of the effective potential for the two geometries. Our calculations have shown that the captured charge is more strongly localized closest to the surface for fullerenes than for cylindrical nanotube.

## 1. Introduction

The experimental and theoretical study of carbon is currently one of the most prevailing research areas in condensed matter physics. Forms of carbon include several allotropes such as graphene and graphite as well as the fullerenes, which cover any molecule composed entirely of carbon, in the form of a hollow sphere, ellipsoid, or tube. Like graphite, fullerenes are composed of stacked graphene sheets of linked hexagonal rings. For these, the carbon atoms form strong covalent bonds through hybridized sp^2^ atomic orbitals between three nearest neighbors in a planar or nearly planar configuration.

Mass spectrometry experiments showed strong peaks corresponding to molecules with the exact mass of sixty carbon atoms and other carbon clusters such as C_70_, C_76_, and up to C_94_ [1, 2]. Spherical fullerenes, well known as “buckyballs” (C_60_), were prepared in 1985 by Kroto et al. [3]. The structure was also identified about five years earlier by Iijima [4], from an electron microscope image, where it formed the core of a multishell fullerene or “buckyonion.” Since then, fullerenes have been found to exist naturally [5]. More recently, fullerenes have been detected in outer space [6]. As a matter of fact, the discovery of fullerenes greatly expanded the number of known carbon allotropes, which until recently were limited to graphite, diamond, and amorphous carbon such as soot and charcoal. Both buckyballs and carbon nanotubes, also referred to as buckytubes, have been the focus of intense investigation, for their unique chemistry as well as their technological applications in materials science, electronics, and nanotechnology [7].

Recently, the image states of metallic carbon nanotubes [8] and double-wall nonmetallic nanotubes [9, 10] were investigated. Experimental work [11] includes photoionization [12] and time-resolved photoimaging of image-potential states in carbon nanotubes [13]. There has been general interest [14] in these structures because of electronic control on the nanoscale using image states. This has led to wide-ranging potential applications including field ionization of cold atoms near carbon nanotubes [15] and chemisorption of fluorine atoms on the surface of carbon nanotubes [16]. The important role, played by the centripetal term in determining the total potential of a captured charged particle, orbiting about C_60_, was demonstrated by McCune et al. in [12]. The local density approximation was used in the calculations of photoionization in that study.

**Figure 1:**
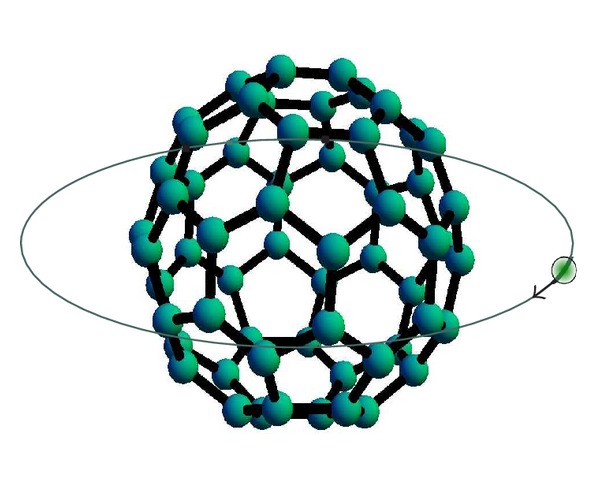
Schematic illustration of a charged particle captured by the image potential and orbiting around a buckyball. The radius of the orbit is determined by the dielectric constant within and surrounding the shell as well as the angular momentum quantum number of the captured particles. For semiconducting shells, the localization is strong and the radius of the stable orbit can be a few nanometers. The localization is weak for metallic shells.

Here, we calculate the nature of the image-potential states in a spherical electron gas (SEG) confined to the surface of a buckyball in a similar fashion as in the case of a nanotube. In Figure 1, we show a schematic of a charged particle localized in a spherical image state. For the semi-infinite metal/vacuum interface [17], related image-potential states have been given a considerable amount of theoretical attention over the years. Additionally, these states have been observed for pyrolytic graphite [18] and metal-supported graphene [19]. Silkin et al. [20] further highlighted the importance of image states in graphene [21] by concluding that the interlayer state in graphite is formed by the hybridization of the lowest image-potential state in graphene in a similar way as it occurs in bilayer graphene [22, 23]. The significance of image states was also discussed by Rinke et al. in [24]. We mention these facts to show why we were motivated to study image states for the spherical geometry. Furthermore, the significance of the role played by the image-potential has led to the observation that for planar layered materials, strongly dispersive interlayer states are present. However, the eigenstates for a spherical shell are nondispersive and so too are the collective plasma modes, [25–29]. This difference in itself leads to unique and interesting properties which we have found for the image potential.

We will consider the image states outside the SEG in the following. However, our method may be extended in a straightforward manner to the case when the image states are inside the shell. A relevant discussion of image states for C_60_ with multiply charged anions [30, 31] was recently given in [32]. Here, we only deal with the case of a singly charged particle. In our formalism for obtaining a spherical image state, we consider a spherical shell of radius *R* whose center is at the origin. The background dielectric constant is *ϵ*_1_ for 0 < *r* < *R* and *ϵ*_2_ for *r* > *R*. An electron gas is confined to the surface of the sphere. If a charge *Q* is located at (*r*_0_, *θ*_0_, *ϕ*_0_) in spherical coordinates, then for *r*_0_ > *R*, the total electrostatic potential is given by Φ_tot_ = Φ_ext_ + Φ_ind_, where Φ_ext_ is the external potential due to the point particle and Φ_ind_ is the induced potential. When *r* < *R*, we express the total potential as follows:

(1) $$\begin{array}{c} \Phi_{\text{tot}}^{\left(1\right)}\left(r,\theta ,\phi \right)=4\pi kQ\underset{LM}{\sum }\frac{1}{2L+1}A_{L}r^{L}Y_{LM}\left(\Omega \right),\\ \Phi_{\text{ext}}\left(r,\theta ,\phi \right)=4\pi kQ\underset{LM}{\sum }\frac{1}{2L+1}\frac{r_{>}^{L}}{r_{>}^{L+1}}Y_{LM}^{*}\left(\Omega_{0}\right)Y_{LM}\left(\Omega \right),\\ \Phi_{\text{ind}}^{\left(2\right)}\left(r,\theta ,\phi \right)=4\pi kQ\underset{LM}{\sum }\frac{1}{2L+1}B_{L}r^{-(L+1)}Y_{LM}\left(\Omega \right), \end{array}$$

where *k* = (4*πϵ*_0_)^−1^ with *ϵ*_0_ the permittivity of free space. Also, *Y*_*LM*_(*Ω*) is a spherical harmonic and *Ω* is a solid angle. For *r* > *R*, the total potential is

(2) Φtot(2)(r,θ,ϕ) =4πkQ∑LM12L+1      ×[r<Lr>L+1YLM∗(Ω0)+BLr−(L+1)]YLM(Ω).

On the surface of the sphere, the boundary conditions are Φ_tot_^(1)^(*R*, *θ*, *ϕ*) = Φ_tot_^(2)^(*R*, *θ*, *ϕ*) and [*ϵ*_1_Φ_tot_^(1)^(*r*, *Ω*; *ω*) − *ϵ*_2_Φ_tot_^(2)^(*r*, *Ω*; *ω*)]|_*r*=*R*_′ = 4*πkσ*(*R*, *θ*, *ϕ*; *ω*) where *σ*(*R*, *θ*, *ϕ*; *ω*) is the induced surface charge density on the spherical shell. Expanding *σ* in terms of spherical harmonics and using linear response theory, we find

(3) $$\begin{array}{c} \sigma \left(R,\theta ,\phi ;\omega \right)=-\frac{2kQe^{2}}{R^{2}}\underset{LM}{\sum }\frac{1}{2L+1}A_{L}R^{L}\Pi_{L}\left(\omega \right)Y_{LM}\left(\Omega \right). \end{array}$$

In this equation, Π_*L*_(*ω*) is the SEG polarization function for *L* an integer and given in terms of the Wigner 3-*j* symbol [25, 27]:

(4) ΠL(ω)=∑ℓℓ′f0(Eℓ)−f0(Eℓ′)ħω+Eℓ′−Eℓ(2ℓ+1)(2ℓ′+1)  ×(ll′L000)2,

where *f*_0_(*E*) = 1/(1 + exp⁡[(*E* − *μ*)/*k*_*B*_ *T*]) is the Fermi-Dirac distribution function, *k*_*B*_ is Boltzmann's constant, and *μ* is the chemical potential. *E*_*ℓ*_ = *ħ* ^2^ *ℓ*(*ℓ* + 1)/(2m*R ^2^) with *ℓ* = 0,1, 2,… and m* is the electron effective mass. The induced potential Φ_ind_^(2)^(**r**; *ω*) outside the spherical shell may be calculated to be

(5) Φind(2)(r;ω) =4πkQ∑L[ϵ2εL(ω=0)−12L+1]R2L+1r0L+1      ×1rL+1∑MYLM∗(Ω0)YLM(Ω),

where *ε*_*L*_(*ω*) = *L*(*ϵ*_1_ + *ϵ*_2_) + *ϵ*_2_ + (2*e* ^2^/*R*)Π_*L*_(*ω*) is the dielectric function of the SEG. The force on a charge *Q* at **r**_0_ = (*r*_0_, *ϕ*_0_, *θ*_0_) is along the radial direction and can be found using *F*(*r*_0_) = −*Q*∂Φ_ind_^(2)^(**r**)/∂*r*|_**r**_0__ yielding

(6) $$\begin{array}{c} F\left(r_{0}\right)=kQ^{2}\underset{L}{\sum }\left(L+1\right)\left[\left(2L+1\right)\frac{\epsilon_{2}}{\varepsilon_{L}\left(\omega =0\right)}-1\right]\frac{R^{2L+1}}{r_{0}^{2L+3}}. \end{array}$$

The interaction potential energy *𝒰*_im_(*r*_0_) may now be calculated from

(7) 𝒰im(r0)≡∑L𝒰im(L)(r0)=12QΦind(r0,θ0,ϕ0)=kQ22r0∑L[(2L+1)ϵ2εL(ω=0)−1](Rr0)2L+1

The effective potential is the sum of the image potential and the centrifugal term and is given by [8, 9]

(8) $$\begin{array}{c} V_{\text{eff}}^{\left(L\right)}\left(r_{0},\theta \right)={𝒰}_{\text{im}}^{\left(L\right)}\left(r_{0}\right)+\frac{\hbar^{2}\left(L^{2}+L-\left(1/4\right)\right)}{2M^{*}{\left(r_{0}\sin\theta_{0}\right)}^{2}} \end{array}$$

showing that *V*_eff_^(*L*)^ is not spherically symmetric. In this notation, M* is the effective mass of the captured charged particle in an orbital state with angular momentum quantum number *L*.

**Figure 2:**
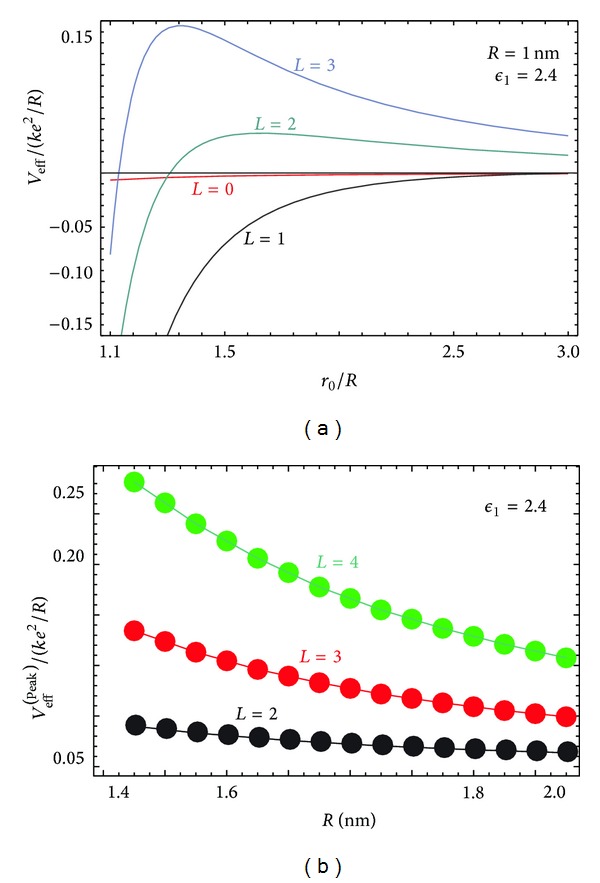
(a) The effective potential *V*_eff_ between a charged particle and a spherical shell is shown for a number of angular momenta *L*. The radius of the sphere is *R* = 1 nm and we chose *ϵ*_1_ = 2.4, *ϵ*_2_ = 1. In (b), the height of the peak in the effective potential appearing in (a) is plotted as a function of the radius.

We now turn to numerical calculation of the effective potential and its comparison with nanotubes. In Figure 2(a), we calculated *V*_eff_^(*L*)^(*r*_0_, *θ*) as a function of *r*_0_, for chosen *L*, *R*. For the background dielectric constant, we chose *ϵ*_1_ = 2.4, corresponding to graphite, and *ϵ*_2_ = 1 for the surrounding medium. The electron effective mass used in calculating the polarization function Π_*L*_ in (4) was *m* = 0.25*m*_*e*_ where *m*_*e*_ is the bare electron mass, the Fermi energy is *E*_*F*_ = 0.6 eV, and the orbiting particle effective mass is M* = *m*_*e*_.

**Figure 3:**
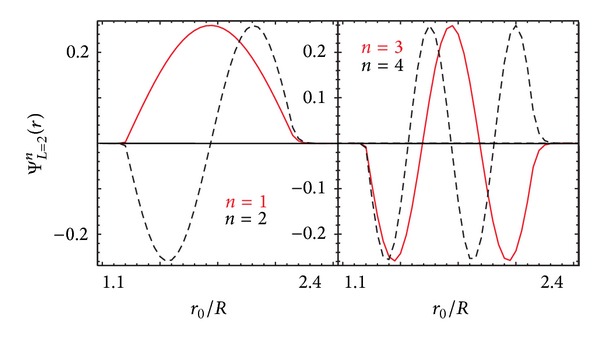
The wave functions for the ground state (*n* = 1) and first three excited states (*n* = 2,3, 4) are plotted for the effective potential *V*_eff_ between a charged particle and a spherical shell when *L* = 2. We chose *ϵ*_1_ = 2.4, *ϵ*_1_ = 1, and *R* = 1 nm.

Figure 2(b) shows how the peak values of the effective potential depend on radius. Of course, the height of the peak is linked to the localization of the particle in orbit. In Figure 3, the ground and three lowest excited state wave functions are plotted for the effective potential *V*_eff_^(*L*)^ when *L* = 2 in Figure 2(a).

The value of the angular momentum quantum number *L* as well as the curvature of the surface of these complex carbon structures clearly plays a crucial role in shaping the effective potential. Generally, the form for the *L*th term may be expressed as *V*_eff_^(*L*)^(*r*_0_) = −*α*_*L*_ *r*_0_^−2(*L*+1)^ + *β*_*L*_ *r*_0_^−2^, where *α*_*L*_ and *β*_*L*_ are due to the image potential and centrifugal force, respectively. The coefficient *α*_*L*_ is always positive, whereas *β*_*L*_ is only negative for *L* = 0. This power-law behavior ensures that no matter what values the two coefficients may have, the image term dominates the centrifugal term, leading to a *l* local maximum in the effective potential. This is unlike the behavior for a cylindrical nanotube where the image term is logarithmic, due to the *linear* charge distribution, and may be dominated by the *r*_0_^−2^ centrifugal term, leading to a local minimum instead. Consequently, the capturing and localization of a charged particle by the image potential of spherical conductors and dielectrics are fundamentally different from those for a cylindrical nanotube. For the sphere, as shown in Figure 3, the wave function is more localized around the spherical shell within its effective potential; that is, the wave function is not as extended. Additionally, the confinement of the charged particle is close to the spherical surface.

The choice for the radius does render some crucial changes in *V*_eff_^(*L*)^ to make a difference in the location and height of the peak. However, only the higher-lying localized states are affected. In Figure 2, we show how the peak height changes as *L* is varied. Additional numerical results corresponding to *ϵ*_1_ ≫ *ϵ*_2_ have shown that a spherical metallic shell has a reduced potential peak for confining the captured charge. Thus, the spherical metallic shell is not as susceptible for particle confinement in highly excited states as the metallic nanotube [8–10]. This indicates that the dimensionality plays a nontrivial role in formation of image states and their spatial extension near the surface of the nanometer-size graphitic structure. This direct crossover from a one-dimensional to a three-dimensional regime is not determined by polarization effects for the structure in the metallic limit since in the limit *ϵ*_1_ → *∞* in (6), the Π_*L*_(*ω*) term makes no contribution. The difference is due entirely to the geometrical shape in the metallic regime where graphitic plasmons fail to develop. For finite values of *ϵ*_1_, we encounter the regime where excited particles contribute through the polarization function Π_*L*_(*ω*) defined in (4). The behavior of plasmon excitation as a function of angular momentum quantum number *L* for fullerenes resembles in all respects the long wavelength (*q* → 0) limit of carbon nanotubes [33–35]. Furthermore, in the case of the low-frequency *π*-plasmons in carbon nanotubes, a surface mode may develop for large *q*, due to the difference in the values for the dielectric constants within the graphitic structure and the surrounding medium [36].

Since the polarization function Π_*L*_(*ω*) vanishes identically for *L* = 0, the attractive part of the effective potential is only significantly modified by screening for a fast-rotating external charge. This behavior at zero angular momentum differs from tubular-shaped image states for single-walled carbon nanotubes which are formed in a potential isolated from the tube [8]. The large angular momentum image states for spheres may be probed by femtosecond time-resolved photoemission [13]. Our formalism shows that considering photoionization from various levels of C_60_, the Coulomb interaction between an external charge and its image is screened by the statically stretched SEG through the dielectric function *ϵ*_*L*_(*ω* = 0). The polarization of the medium Π_*L*_ which is driven by the electrostatic interaction is generated by particle-hole transitions across the Fermi surface. The polarization also determines the Ruderman-Kittel-Kasuya-Yosida (RKKY) interaction energy between two magnetic impurities as well as the induced spin density due to a magnetic impurity.

Increasing radius, the position of the peak moves closer to the sphere as *R* ^−1/(2*L*)^. In the absolute units, the position of the peak depends as *r*_0_^(peak)^∽*R* ^1−1/(2*L*)^. The typical distances from the surface are between 1.3*R* and 1.5*R*. Figure 2(b) demonstrates how the potential peaks (corresponding to the local maximum for the *V*_eff_) depend on the radius of the buckyball for various angular momentum quantum numbers *L*. Clearly, we see that the potential peak decreases with increased radius leading us to conclude that confinement is the strongest for smaller buckyballs and particles with large angular momentum. For the nanotube, increasing *L* leads to a reduced local minimum in the effective potential and the ability to localize the charge [8]. Approximately, the curves may be fitted analytically to ∽1/*R* for all considered values of *L*. However, we found that a better fit for *L* > 5 would be of the form *c*_1_/*R* + *c*_2_/*R* ^2^ where *c*_1_, *c*_2_ are constants.

For increased *ϵ*_1_, that is, the metallic limit with *ϵ*_1_ ≫ *ϵ*_2_, we have *ϵ*_*L*_⋍*ϵ*_1_ *L*, so that the coefficient [(2*L* + 1)(*ϵ*_2_/*ϵ*_*L*_) − 1] → −1. In the case of dielectric constant *ϵ*_1_ ~ 2.4 for the buckyball, the above-mentioned coefficients lie within the range from −0.4 to −0.9 and decrease with increasing *L*. Consequently, for the transition to the metallic limit, these coefficients are more affected for states with large *L*. So, we may conclude that *ϵ*_1_ has little effect on the position and height of the peak in the effective potential. In fact, for the metallic case, the peak is observed to be slightly further away from the center (very little difference ~1.37*R* compared to ~1.31*R* for *R* = 1 nm). The height of the peak is only slightly decreased in the metallic case (0.24 compared to 0.27 in the case of fullerenes). These numbers are provided for fixed *L* and the unit of energy is the same as that in Figure 2.

**Figure 4:**
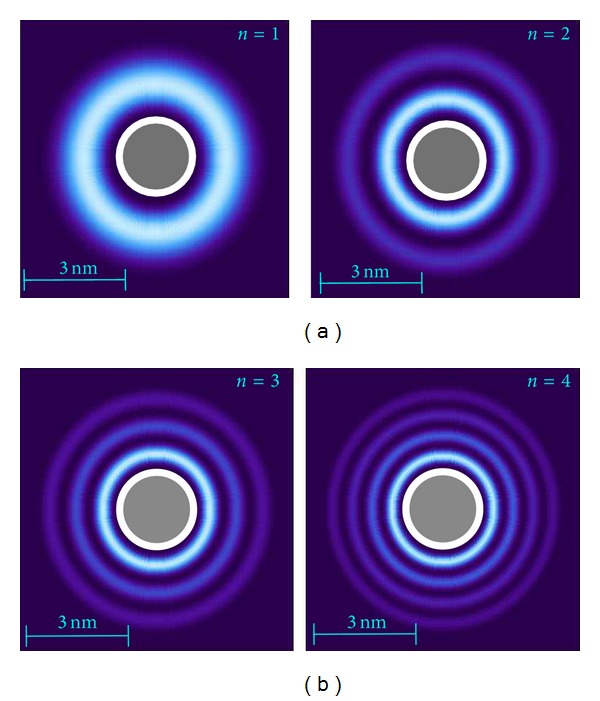
Probability density plots for |Ψ_*L*,*n*_(*r*_0_)|^2^/*r*_0_^2^ when *L* = 2 and *n* = 1,2 (a) as well as *n* = 3,4 (b), where *n* labels the eigenstates, for a spherical shell of radius *R* = 10 Å. The wave function Ψ_*L*,*n*_(*r*_0_) is a solution of the one-dimensional Schrödinger equation with effective potential *V*_eff_(*r*_0_, *θ* = *π*/2) shown in Figure 2. We chose *ϵ*_1_ = 2.4 inside the ball, whose outline is shown as a thin circle, and *ϵ*_2_ = 1 in the surrounding medium.

Regarding the wave functions and density plots, Figures 3 and 4 demonstrate the wave function of a bounded electron trapped between the infinite hard wall of the sphere and the potential peak. First, we note that we obtained qualitatively similar behavior for different values of *L*, so the electron states corresponding to the potentials with different angular momenta are almost the same. We clearly see that the electron wave functions are not exactly localized in the “potential well” due to the asymmetry of the boundary conditions, that is, infinitely high wall on the left and the effective potential profile on the right-hand side. The wave functions corresponding to *L* = 0 are extremely delocalized due to the relatively shallow potential. The fact that the effective potential is not spherically symmetric means that, for arbitrary angle *θ*, we must solve a three-dimensional Schrödinger equation. However, for trajectories parallel to the *x* − *y* plane for the constant angle *θ*, the problem reduces to a quasi-one-dimensional Schrödinger equation involving the radial coordinate. In our calculations, we set *θ* = *π*/2 so that the captured charge is moving in the equatorial plane. In this case, the centrifugal term is weakest compared to the image potential, but it still affords us the opportunity to see its effect on localization.

The density plots in Figure 4 show that the innermost ring is substantially brighter than the outer rings. This is a consequence of the presence of the *r*_0_^−2^ factor in the electron probability function. In contrast, the corresponding plots for the nanotube [10] do not have the innermost ring so much brighter than the outer rings because of the fact that the density function in that case depends on the inverse distance of the charge from the center of the cylinder instead. This is another unusual, specific feature of the considered geometry and indicates that the captured charge is more strongly localized for the spherical shell closest to the surface for fullerenes than for cylindrical nanotube. The lowest bound states for Figure 3 are in the range from −10 to about −100 meV, with the first few excited states lying very close to the ground state energy.

In conclusion, we note that our calculations have shown that the bound state energies of charged particles, localized around nanosized spherical shells such as buckyballs, may be adjusted by varying the radius *R* of the shell. In our paper, we used an electron gas model for the electron energies. However, we may incorporate a more realistic energy band structure into the polarization function through a form factor by making use of the results presented in [37]. This would also account for the prescribed number of electrons on the fullerene. We note that the peak potential decreases according to a power-law function with increasing radius. This property allows for considerable manipulation of a captured external electron and its release to a source of holes for recombination followed by the release of a single photon whose frequency and polarization are linked to those of the electron. This single-photon source may have variable frequency with a broad range of applications in quantum computation where the message is encoded in the number of photons transmitted from node to node in an all-optical network. Gate operations are performed by the nodes based on quantum interference effects between photons which cannot be identified as being different. The low frequency photons could be in the infrared, which is the most useful range for telecommunications. Another, more general, practical application and technological use of such unique quantum states would be to quantum optical metrology of high accuracy and absolute optical measurements.
